# The potential impacts of regional artificial intelligence development on depressive symptoms in older adults: evidence from China

**DOI:** 10.3389/fpsyg.2026.1781672

**Published:** 2026-02-17

**Authors:** Shenwei Wan, Yixiao Liang, Zhiwen Ding, Yong Tang, Liangshan Yang

**Affiliations:** 1 Institute of Rural Development, Zhejiang Academy of Agricultural Sciences, Zhejiang, China; 2 School of Agricultural Economics and Rural Development, Renmin University of China, Beijing, China; 3 School of Economics and Management, Northeast Forestry University, Harbin, China; 4 Institute of Agricultural Information, Chinese Academy of Agricultural Sciences, Beijing, China

**Keywords:** artificial intelligence, depressive symptoms, heterogeneity, older adults, two-way fixed effect

## Abstract

Depression is increasingly prevalent among older adults worldwide, exacerbated in the post-pandemic era and driven by aging populations, economic strain, and quality-of-life declines. In China, these factors contribute significantly to arise in depression among this demographic. Meanwhile, Artificial Intelligence (AI) shows growing promise in mental health management, potentially offering valuable tools to mitigate depression. This study examines AI’s capacity to alleviate depressive symptoms in older adults from a macroeconomic perspective, particularly in aging societies like China and other developing nations. Using data from the China Health and Retirement Longitudinal Study (CHARLS) spanning 2011–2020, employ a two-way fixed-effects model to empirically analyze AI’s impact on depression in this demographic. Our results indicate a significant negative association between AI development and depressive symptoms among older adults. Mediation analysis reveals that macroeconomic factors, such as increased Internet access, robot application density, and investment in science and technology, and micro-level factors, like life satisfaction and cognitive function, contribute to AI’s beneficial impact on mental health. While our findings are robust, limitations include data constraints and the need for further exploration of specific AI applications on depression outcomes. Future research could focus on interdisciplinary approaches integrating AI with psychomedical technologies, emphasizing support for vulnerable groups, including those in rural or under-resourced areas, and fostering public awareness and accessibility of AI health tools.

## 1 Introduction

Depressive symptoms have become a prevalent mental health challenge among older adults globally (Koenig et al., 2020). It is estimated that 5% of adults worldwide suffer from depressive disorders each year (Herrman et al., 2022). Most studies indicate a general upward trend in the global prevalence of depression in recent years, particularly in the post-pandemic era (Donnelly and Farina, 2021; Ran et al., 2020; Zheng et al., 2021). In many nations, population aging and widening socio-economic inequalities have further amplified the mental health burden on older adults. In China, specifically, the prevalence of depression among this demographic has risen significantly, driven by rapid demographic shifts and increasing vulnerability in later life (Fei et al., 2018; Santomauro et al., 2021; Zhao et al., 2020). Older adults in China face multifaceted challenges, including economic downturns, declining quality of life, and persistent age-related stereotypes that may discourage help-seeking and limit access to appropriate care. The rise in mental health problems among this population not only affects individual well-being but also imposes a significant socio-economic burden by increasing healthcare costs (Hou and Liu, 2025; Miller et al., 2014). Therefore, there is an urgent need—both in China and globally—to explore effective strategies to prevent and alleviate depression, ensuring mental health management is adapted to the current era of rapid population aging.

Concurrently, the rapid advancement of Artificial Intelligence (AI) has attracted growing international attention as a transformative tool for mental healthcare systems. AI advancements have already been translated into clinical practice (Park et al., 2024), and health management aids such as remote diagnostics and wearable devices are gaining prominence at the forefront of medicine. In the field of mental health, AI-assisted interventions facilitate early detection and support timely treatment (Marwaha et al., 2016; Vo et al., 2023). The convergence of AI and healthcare is reshaping the future of medical systems (Alhuwaydi, 2024; Sharma et al., 2023; Tornero-Costa et al., 2023), presenting new opportunities to alleviate depression among older adults. However, international debates also highlight that digital innovations may unintentionally reproduce or exacerbate existing forms of social exclusion and ageism, particularly if older adults are under-represented in training datasets or face structural barriers to accessing digital services.

A growing body of scholarship has examined the relationship between AI and depressive symptoms, though with varying focuses. Geographically, existing studies have primarily concentrated on developed countries. For example, researchers utilizing AI deep learning tools alongside telephone interviews in eight US states found significant impacts of depression on the general population (Bone et al., 2022; Edwards et al., 2019; Ohayon et al., 2023). Similarly, analyses of Biobank data have investigated the relationship between environmental factors (e.g., PM2.5 exposure) and major depression, identifying vitamin D and physical activity as mitigating factors (Wu et al., 2022). Regarding study populations, existing work has often focused on adolescents. Studies employing diagnostic interviews with adolescents aged 13 to 16 have proposed effective screening tools (Ma et al., 2022; Pietsch et al., 2011), noting that adolescent depression may elevate the risk of depression in adulthood (Johnson et al., 2018). In terms of research content, many studies focus on the technical application of AI, such as clinical decision support tools, algorithms, and telemedicine implementation (Luo et al., 2022), or conduct proof-of-concept studies using machine learning to explore early treatment options (Salem et al., 2023).

Despite this progress, existing studies exhibit several notable limitations from both a global health and an age-inclusive perspective. First, there is a lack of specific focus on depression among older adults in developing countries. Methodologically, prior research has frequently neglected the heterogeneity arising from regional, cultural, and socio-economic disparities. Mental health management models in developed countries typically depend on advanced infrastructure and abundant resources, which are challenging to replicate in developing settings constrained by limited funding and professional shortages. These structural disparities intersect with ageism: older adults may have fewer opportunities to participate in digital health initiatives. Furthermore, current AI prediction models are predominantly trained on data from Western populations, potentially failing to capture risk factors specific to the Chinese context—such as family structure and social pressure (Wei and Li, 2022). Consequently, existing models often overlook these critical cultural nuances. Second, while most research emphasizes micro-level technological applications, there is a dearth of in-depth analysis regarding AI’s broader impact on the overall mental health burden and the sustainability of mental health management from a health economics perspective. This gap is particularly salient in aging societies, where policy decisions significantly shape the distribution of mental health resources.

To address these gaps, this study empirically analyzes the potential of regional AI development in addressing depression among older adults from a health economics perspective. By incorporating specific control variables—including family size, household income, and family support—we aim to develop an analysis that better reflects the lived realities of older adults in China. This paper examines how AI innovations can help prevent and reduce depressive symptoms and promote the sustainable development of mental health management, while also contributing to broader international debates on healthy aging and the reduction of age-related inequalities.

## 2 Theoretical logic and research hypothesis

The rapid development of artificial intelligence (AI) has had a profound impact on multiple dimensions of global society. For older adults in China and other rapidly aging societies, AI has the potential to improve quality of life and alleviate some of the structural and psychosocial pressures they face. From a macro perspective, AI can drive economic growth, strengthen regional infrastructure, and accelerate the flow of social capital, thereby providing a stronger contextual foundation for the mental health of older adults. Moreover, AI-supported health systems can offer this population more diverse, person-centered, and personalized healthcare services (Kim et al., 2023; Mu et al., 2023). From a micro perspective, AI technologies can augment the capabilities of older adults, facilitate their participation in social activities (Char et al., 2018), and enable them to receive psychological support and medical care in more timely and flexible ways, thereby helping to reduce the incidence of depressive symptoms. Based on the above analysis, we propose the following research hypothesis:

*H*1: AI development is negatively correlated with depressive symptoms in older adults.

At the macro level, the alleviating effect of AI industry development on depressive symptoms among older adults is primarily mediated through several key mechanisms. First, AI development accelerates social and economic modernization. Economic modernization has heightened the demand for efficient information exchange, fostering the widespread adoption and advancement of Internet technologies. These developments have improved convenience for older adults in everyday domains such as payments, shopping, and travel. In the medical sector, innovations like online appointment systems and Internet-based healthcare services have effectively mitigated challenges such as limited access to healthcare and long waiting times for this demographic. At the same time, Internet technologies can narrow the information gap between older adults and the rest of society, reduce perceptions of social exclusion, and thereby lower the risk of depression (Coventry et al., 2014; Dicuonzo et al., 2023), provided that digital tools are designed and implemented in an age-inclusive manner. Second, AI development has spurred greater adoption of automation technologies by enterprises, elevating automation levels within technology firms and significantly boosting regional productivity. This process has expanded the variety and accessibility of goods and services available to older adults, enriching their quality of life (Johnson et al., 2021). Third, the growth and concentration of the AI industry have attracted substantial capital investment from both government and private sectors, stimulating regional GDP growth. This economic uplift can increase the income of older adults and help alleviate financial strain and everyday life pressures (Dong et al., 2024). Based on the above analysis, we propose the second research hypothesis:

*H*2: AI development has the potential to alleviate depressive symptoms in older adults by increasing the Number of Internet accesses, the Density of robot applications, and the Investment in science and technology.

At the micro level, the mitigating effect of AI industry development on depressive symptoms among older adults is primarily manifested through the following pathways. First, AI development has improved access to public services and social resources for this demographic. Compared with the material scarcity that many experienced in their youth and early adulthood, the current abundance of material and digital resources—when made accessible to older adults—can substantially enhance life satisfaction (Ali et al., 2023). Second, AI-enabled technologies provide older adults with diverse channels for social interaction and information acquisition, including social media, online communities, and intelligent communication tools. These technologies facilitate a more comprehensive understanding of contemporary society, increase social participation, and promote cognitive engagement. In turn, they may enhance older adults’ sense of social achievement and cognitive functioning, thereby buffering against depressive symptoms (Danieli et al., 2021; Rathnayaka et al., 2022). Based on the above analysis, we propose the third research hypothesis:

*H*3: AI development can alleviate depressive symptoms in older adults by improving their life satisfaction, enhancing their social achievement, and boosting their total cognitive level.

Drawing on the Cobb–Douglas economic model, higher investments in fixed assets and labor, given a relatively fixed level of land capital, are associated with greater industrial development (Cheng and Han, 2014). In the context of this study, the total investment in fixed assets and human capital within the tertiary industry of the prefecture-level city where the sample is located serves as a catalyst for the growth of the emerging AI industry. Such investments not only enhance the efficiency of AI technology innovation and application but also facilitate the wider dissemination of AI-related products and services to older adults. This expanded reach can improve access to psychological support and emotional care for this population, thereby helping to alleviate depressive symptoms. Based on the above analysis, we propose the fourth research hypothesis:

*H*4: The greater the total fixed asset investment and employment in the tertiary industry within the prefecture-level city where the sample is located, the more pronounced the mitigating effect of AI development on the depressive symptoms of older adults.

## 3 Study design and methods

### 3.1 Data sources

China Health and Retirement Longitudinal Study (CHARLS)^1^ is a nationwide demographic survey in China that adopts a multistage cluster sampling method and conducts face-to-face interviews with the sample, obtaining comprehensive information on various aspects of the sample, including individual mental health. The baseline survey was conducted in 2011, covering 150 district-level units, 450 village-level units, and 17,000 people in about 10,000 households, with a good overall response rate that aligns with the economic structural characteristics and ethical requirements of developing countries. After removing outliers and interpolating missing values (removing questionnaire data where all answers submitted by participants were the same), this paper uses data from the five-period (2011, 2013, 2015, 2018, and 2020) CHARLS surveys and data from the China Municipal Statistical Yearbook, totaling 10,104 older adults aged 60 and older across 5 years. This yields a total of 50,520 panel data samples. While constructing a balanced panel may introduce attrition bias, it likely leads to conservative estimates because dropouts in longitudinal aging studies are often associated with poorer health or mortality. Thus, any observed positive association between AI development and mental health in this “survivor” sample suggests the effect is robust.

### 3.2 Variable selection

#### 3.2.1 Assessment of the depressive symptom of older adults

This article measures depressive symptoms in older adults based on the final scores of the CES-D-10 scale from the CHARLS questionnaire (cesd10), which transforms character variables into multivalued numeric variables with a total score ranging from 0 to 30; higher scores are associated with more severe depressive symptoms, while lower scores are associated with less severe depressive symptoms.

#### 3.2.2 Assessment of the artificial intelligence advancement

In measuring the AI development level, we refer to previous research (Sun et al., 2024), based on the AI development level data at the prefecture-level city level in China, identified using a feature keyword extraction method, and based on the economic scope of each enterprise published in the TianYanCha’s information database for text analysis. When organizing the data, we focused on the business scope of the enterprise. If the business scope of the enterprise involves keywords related to artificial intelligence such as chips, image recognition, computer vision, speech recognition, sensors, etc., the enterprise will be identified as an artificial intelligence enterprise. We selected 300 prefecture-level cities with more than 10,000 samples as the original data, resulting in five periods of samples for the 2011, 2013, 2015, 2018, and 2020 for the AI development level of prefecture-level cities, matched one by one with the micro-samples. At the same time, to minimize the effects of heteroskedasticity, this paper applies logarithmic transformations to the number of AI enterprises in the sample’s region to measure the local AI development level. While enterprise density is a macro-level industrial indicator, it effectively proxies the regional availability of AI-enabled resources. Following the logic of technological spillovers, high-density AI clusters typically foster a “Smart City” ecosystem, translating industrial capacity into accessible public goods—such as telemedicine platforms, smart community monitoring, and automated care services—that directly benefit the elderly population.

#### 3.2.3 Assessment of moderator variables

Considering the previous analysis of the moderating mechanism between the Cobb–Douglas economic model and the level of AI development, drawing on existing literature (Cheng and Han, 2014),we selected the total fixed asset investment (in millions of yuan) and tertiary employment (in tens of thousands) from the sample’s prefectural cities as the moderating variables at the capital and labor force levels, respectively. By establishing the set of fixed asset investment indicators and employment indicators of the tertiary industry and the interaction term of explanatory variables, we characterize the mediating role of fixed asset investment and employment in the process of artificial intelligence’s impact on the depressive symptoms mental health of older adults.

#### 3.2.4 Assessment of moderator covariates

Considering the previous analysis of the mechanism through which AI development affects depressive symptoms in older adults, and drawing on existing literature (Luo et al., 2022), we selected life satisfaction (satlife), social achievement (soc), and total cognitive level (total_cognition) as micro-level mediating variables of the effect of AI development on depressive symptoms among older adults. These variables were derived from the CHARLS questionnaire and the US Health and Retirement Study (HRS). At the macro level, we selected the number of Internet accesses (num_net), density of robot applications (bot), and investment in science and technology (tech) as mediating variables of AI development’s impact on depressive symptoms in older adults.

#### 3.2.5 Assessment of covariates

In terms of covariates, we mainly refer to the existing literature (Dong et al., 2023; Qiao et al., 2022), and select four individual-level variables: age (age), gender (gender), education (edu), and marital status (marry). At the household level, we include family size (family_size) and the number of children (hchild). At the regional level, we use three variables: the Gross Domestic Product (gdp), the total population (pop), and the urbanization rate (cityrate) of the sample’s prefecture-level city. These eight covariates are included to minimize the potential endogeneity problems caused by omitted variables.

### 3.3 Model setting

Given that the CHARLS dataset comprises five periods of panel data, unobservable geographic and time-specific factors may influence the results. To determine the most appropriate model for our analysis, we conducted the Hausman test to compare the fixed effects and random effects specifications. The test yielded a statistically significant result (*p* < 0.01), rejecting the null hypothesis that the random effects estimator is consistent, indicating that the random effects model’s assumption of no correlation between the unobserved effects and the explanatory variables does not hold. This suggests that the fixed effects approach is more consistent for our data. To minimize model error and improve estimation accuracy, we employ a two-way fixed-effects model as the baseline regression, which is specified as follows:


(1) $$C_{\mathrm{it}}=\alpha +\beta_{1}A_{\mathrm{it}}+\sum_{n}^{j=1}v_{j}V_{\mathrm{it}}+\mu_{i}+\lambda_{t}+\varepsilon_{\mathrm{it}}$$

In Equation 1, $C_{\mathrm{it}}$ denotes the depressive symptoms of older adults in year t of the ith district (cesd10), and $A_{\mathrm{it}}$ is the level of development of AI (AI) of the prefecture-level city where this sample is located, the coefficient $\beta_{1}$ of $C_{\mathrm{it}}$ indicates the extent of the effect of depressive symptoms among the elderly in the sample within the prefecture level of AI development in that prefecture level, $V_{\mathrm{it}}$ is a series of control variables, $\mu_{i}$ is an individual-fixed effect, $\lambda_{t}$ is a time-fixed effect, $\varepsilon_{\mathrm{it}}$ is a randomized perturbation term.

## 4 Results and analysis

### 4.1 Descriptive statistical analysis

In this paper, we first analyzed the data with descriptive statistics, using a combined sample size of 50,520 older adults in our study. We used the CES-D-10 score to respond to the level of depression among older adults in the sample at the prefecture-level as an explanatory variable in the regression analysis. The data showed that the mean depression level of older adults was high (8.228 ± 6.293, Mean ± SD), indicating that depression is a widespread issue among older adults. In addition, we chose the logarithm of the number of AI enterprises as the core explanatory variable, and the data show that the mean of the logarithm of the number of AI enterprises in prefecture-level cities in the sample is low (4.551 ± 1.850, Mean ± SD), indicating that AI development in prefecture-level cities is generally low, with some areas lacking AI enterprises. The specific statistical values of other variables are also listed in Table 1.

**Table 1:** Descriptive statistics.

Type	Variable	Definition	Mean	Std. Dev.	Min	Max
Explained variables	cesd10	depressive symptom	8.228	6.293	0	30
Core explanatory variables	AI	Artificial Intelligence advancement	4.551	1.850	0.000	10.110
Mediator variables	satlife	Satisfaction with life	3.226	0.781	1.000	5.000
	soc	Social intensity (mean of 8 types of activity measurements)	0.797	1.013	0.000	8.000
	total_cognition	Total Cognitive Level Score	12.265	3.523	0.000	21.000
	num_net	Number of Internet broadband access households (thousands)	6.537	0.993	3.611	9.139
	bot	Number of industrial robots per 10,000 people	4.789	4.276	0.101	24.652
	tech	The proportion of scientific expenditure to the total local fiscal expenditure (%)	0.016	0.016	0.001	0.140
Moderator variables	invest	Total investment in fixed assets (Ten thousand yuan)	27700000.000	29200000.000	1990000.000	167000000.000
	job	Employment in the tertiary industry (Ten thousand people)	40.785	62.519	7.860	436.460
Covariates	age	Age of respondent	68.420	7.550	60.000	87.000
	gender	Gender of respondent	0.492	0.500	0.000	1.000
	edu	Educational attainment of respondent	0.481	0.500	0.000	1.000
	marry	Marital status of respondent	0.858	0.350	0.000	1.000
	family_size	Number of family members	3.162	1.567	1.000	8.000
	hchild	Number of living children	2.587	1.367	0.000	7.000
	gdp	Urban Economicsituation (Ten thousand yuan)	32800000.000	39000000.000	2900000.000	229000000.000
	pop	Urban population (Ten thousand people)	620.469	440.951	130.900	3371.840
	cityrate	Urbanization rate (%)	0.532	0.139	0.245	0.893

### 4.2 Benchmark regression analysis

Before regression we used variance inflation factor (VIF) to test for multicollinearity between the variables and the results showed that the mean value of VIF was 1.54 and the maximum value was 2.98, indicating that there was no significant multicollinearity. Then we used a two-way fixed-effects model to test the relationship between AI development and the level of depression among older adults. We conducted regression analysis with AI development in prefecture-level cities as the explanatory variable and depression levels of older adults in prefecture-level cities as the dependent variable. To account for potential serial correlation of error terms within regions and avoid Moulton bias, we cluster standard errors at the prefecture-city level in all regression specifications. The results of the benchmark regression are shown in Table 2. Model (1) shows the regression results without any control variables. The results indicate a significant negative correlation between AI progress and depression levels among older adults; Model (2) indicates the regression results that include some control variables but do not account for city fixed effects, meaning the control variables do not capture differences between prefecture-level cities. The results show that the progress of AI is significantly negatively correlated with the level of depression among older adults; Model (3) represents the regression results with control variables included. The results show that the progress of artificial intelligence has a significant negative correlation with depression levels among older adults. Based on the above analysis, hypothesis H1 is established.

**Table 2:** Analysis of baseline regression results.

Variables	(1)	(2)	(3)
	cesd10	cesd10	cesd10
AI	−0.0709***	−0.1085***	−0.0908***
	(0.0144)	(0.0144)	(0.0151)
age		0.0041	0.0068**
		(0.0028)	(0.0029)
edu		−0.8087***	−0.8101***
		(0.0218)	(0.0221)
gender		−1.5687***	−1.5793***
		(0.0460)	(0.0458)
marry		−1.3500***	−1.2986***
		(0.0705)	(0.0701)
family_size		−0.0948***	−0.1061***
		(0.0150)	(0.0152)
hchild		0.1449***	0.1238***
		(0.0197)	(0.0204)
gdp		−0.0000***	−0.0000***
		(0.0000)	(0.0000)
pop		0.0005***	−0.0000
		(0.0001)	(0.0001)
cityrate		−3.8939***	−2.9773***
		(0.2361)	(0.2595)
Constant	8.5548***	13.9275***	13.4969***
	(0.0688)	(0.2455)	(0.2637)
R-squared	0.034	0.080	0.096
CityFE	YES	NO	YES
YearFE	YES	YES	YES

* means *p* < 0.1, ** means *p* < 0.05, *** means *p* < 0.01, and the standard errors in parentheses.

### 4.3 Endogenous treatment

Considering the endogeneity problem between the random error term and the core explanatory variables, we established the Two-Stage Generalized Method of Moment regression model (2stageGMM). To enhance identification strategy and enable validity testing, we employed two instrumental variables simultaneously: the average slope of topography (Liu et al., 2021) and a Bartik (shift-share) instrument.

First, regarding the topographic instrument, using slope satisfies the valid conditions: (1) it is a naturally occurring objective variable independent of socio-economic activities and the random perturbation term; (2) steeper slopes hinder AI infrastructure construction, ensuring a negative correlation with the endogenous variable; and (3) slope does not directly affect older adults’ depression, satisfying exogeneity. Furthermore, by explicitly controlling for regional GDP, population density, and urbanization rate, our model effectively blocks alternative channels (e.g., general economic prosperity or transport accessibility) through which topography might affect mental health, satisfying the exclusion restriction.

Second, we constructed a Bartik shift-share instrument by interacting the city’s initial share of employment in the Information Transmission, Computer Services, and Software industry (base year 2011) with the national annual growth rate of fixed asset investment in the corresponding industry. The rationale is that national industrial trends (the shift) are exogenous to local health conditions, while the initial industrial structure (the share) determines local sensitivity to these trends.

Table 3 reports the results of the instrumental variable tests using both instruments. The first-stage regression shows that the topographic slope is significantly negatively correlated, while the Bartik instrument is significantly positively correlated with the number of AI firms. The Kleibergen-Paap rk Wald F statistic is 24.56, significantly exceeding the conventional threshold of 10, which rules out weak instrument concerns. Crucially, since we employ two instruments for one endogenous variable, we performed the Hansen J overidentification test. The *p*-value of the Hansen J statistic is 0.452, which is well above 0.1, indicating that we cannot reject the null hypothesis that the instruments are valid and uncorrelated with the error term. After mitigating endogeneity, the log of AI firms remains significantly and negatively associated with depressive symptoms in older adults.

**Table 3:** Results of instrumental variable approach (two-stage GMM).

Variables	(4)	(5)
	Topography	Topography + Bartik
AI(second stage)	−0.0193***	−0.0854***
	(0.0066)	(0.0245)
topo(first stage)	−32.366***	−30.125***
	(11.1140)	(9.8500)
Bartik(first stage)		0.6150***
		(0.1520)
Control variables	YES	YES
Kleibergen-Paap LM	8.520***	15.680***
Kleibergen-Paap Wald F	11.140***	24.560***
Hansen J (p-value)		0.452
R-squared	0.016	0.016
CityFE	YES	YES
YearFE	YES	YES

* means *p* < 0.1, ** means *p* < 0.05, *** means *p* < 0.01, and the standard errors in parentheses.

### 4.4 Robustness check

To verify the robustness of our baseline findings, we conducted a series of sensitivity analyses reported in Table 4. First, we replaced the dependent variable with alternative health measures. Model (6) uses a binary indicator of whether older adults had any outpatient visits in the previous year. The results show that regional AI development remains significantly negatively correlated with the likelihood of seeking outpatient care. Second, we utilized the frequency of outpatient visits as the dependent variable in Model (7). The results indicate that regional AI development continues to be significantly negatively correlated with the number of outpatient visits. We acknowledge that outpatient visits primarily reflect healthcare utilization and physical health status rather than depressive symptoms directly. However, these results suggest that AI development is associated with a broader improvement in well-being, leading to a reduced frequency of medical consultations. Third, we employed a panel Tobit model to address the bounded nature of the depression score (Model 8). The results remain consistent, with AI development showing a significant negative correlation with depressive symptoms. This indicates that our findings are not driven by the truncation of the dependent variable. Fourth, we replaced the key explanatory variable (log of AI enterprises) with the actual number of AI enterprises (unlogged). Model (9) shows that the absolute number of AI enterprises is still significantly negatively correlated with depressive symptoms, confirming that the results are not sensitive to the functional form of the independent variable. Finally, to address concerns regarding the incidental parameters problem in nonlinear panel models and to verify the robustness of our results to alternative functional forms, we constructed a binary depression indicator (assigned a value of 1 if the CES-D score > = 10, and 0 otherwise) and estimated a Linear Probability Model (LPM). The results, presented as a supplementary check, show that the coefficient of AI development is −0.0125 (Model (10)), indicating that regional AI advancement is significantly associated with a lower probability of older adults suffering from depression. This finding is consistent with the baseline results derived from the continuous CES-D score.

**Table 4:** Results of Robustness check.

Variables	(6)	(7)	(8)	(9)	(10)
	Replace y with whether outpatient in the previous year	Replace y with the number of outpatient visits in the previous year	Replacement method for panel Tobit model	Replacing x with an unlogged real value	LPM
	doctor	doctor_time	cesd10	cesd10	Depression (Binary)
AI	−0.0029***	−0.0122***	−0.0628***		−0.0125***
	(0.0009)	(0.0039)	(0.0223)		(0.0035)
AI_real				−0.0000***	
				(0.0000)	
age	0.0011***	0.0039***	0.0219***	0.0067**	0.0010**
	(0.0002)	(0.0007)	(0.0040)	(0.0028)	(0.0004)
edu	0.0027**	−0.0128**	−0.7949***	−0.8115***	−0.1120***
	(0.0014)	(0.0058)	(0.0321)	(0.0221)	(0.0250)
gender	−0.0443***	−0.1174***	−1.6565***	−1.5753***	−0.2150***
	(0.0028)	(0.0119)	(0.0725)	(0.0457)	(0.0420)
marry	−0.0014	−0.0214	−1.3481***	−1.3043***	−0.1805***
	(0.0043)	(0.0181)	(0.0902)	(0.0700)	(0.0510)
family_size	0.0017*	−0.0002	−0.0673***	−0.1050***	−0.0145***
	(0.0009)	(0.0039)	(0.0154)	(0.0152)	(0.0050)
hchild	0.0026**	0.0075	0.0888***	0.1292***	0.0168***
	(0.0012)	(0.0052)	(0.0272)	(0.0203)	(0.0060)
gdp	0.0000	−0.0000	−0.0000***	−0.0000***	−0.0000***
	(0.0000)	(0.0000)	(0.0000)	(0.0000)	(0.0000)
pop	−0.0000**	−0.0000	0.0006***	−0.0000	−0.0000
	(0.0000)	(0.0000)	(0.0001)	(0.0001)	(0.0000)
cityrate	−0.0812***	−0.1273*	−3.5537***	−2.8315***	−0.4129***
	(0.0160)	(0.0667)	(0.2922)	(0.2569)	(0.1250)
sigma_u			4.3168***		
			(0.0298)		
sigma_e			4.7411***		
			(0.0152)		
Constant	0.1927***	0.4928***	−295.8405***	13.0361***	0.4520***
	(0.0162)	(0.0674)	(20.0553)	(0.2497)	(0.0124)
R-squared	0.015	0.077	0.017	0.096	0.048
CityFE	YES	YES	YES	YES	YES
YearFE	YES	YES	YES	YES	YES

* means *p* < 0.1, ** means *p* < 0.05, *** means *p* < 0.01, and the standard errors in parentheses.

## 5 Discussion

### 5.1 Mechanism of action analysis

#### 5.1.1 Analysis of micro-level mechanism

Following Jiang (2022), we adopt a two-step approach to test the mediating effects. Table 5 presents the results regarding the role of AI development in alleviating depressive symptoms among older adults through micro-level pathways. The results indicate that regional AI development is significantly and positively associated with older adults’ life satisfaction (Model 10, *p* < 0.01), social achievement (Model 11, p < 0.01), and total cognitive level (Model 12, *p* < 0.01). These findings suggest that AI advancement may alleviate depressive symptoms by enhancing psychosocial well-being and cognitive function, thereby validating the proposed mediating pathways. This aligns with Jaso et al. (2020), yet our study extends this prior work by systematically delineating the specific channels—life satisfaction, social engagement, and cognitive functioning—through which these benefits materialize.

**Table 5:** Analysis of micro-level mechanism results.

Variables	(10)	(11)	(12)
	satlife	soc	total_cognition
AI	0.0078***	0.0107***	0.0292***
	(0.0019)	(0.0024)	(0.0084)
age	0.0057***	−0.0099***	−0.0707***
	(0.0004)	(0.0004)	(0.0016)
edu	0.0008	0.1634***	1.2194***
	(0.0028)	(0.0035)	(0.0119)
gender	0.0344***	−0.0302***	0.3492***
	(0.0058)	(0.0072)	(0.0252)
marry	0.1037***	−0.0347***	0.5771***
	(0.0089)	(0.0108)	(0.0411)
family_size	0.0155***	−0.0149***	−0.0138
	(0.0019)	(0.0024)	(0.0085)
hchild	0.0089***	−0.0194***	−0.1497***
	(0.0026)	(0.0031)	(0.0117)
gdp	−0.0000	0.0000***	0.0000***
	(0.0000)	(0.0000)	(0.0000)
pop	0.0000	−0.0001***	−0.0005***
	(0.0000)	(0.0000)	(0.0001)
cityrate	0.2709***	0.1360***	1.5603***
	(0.0333)	(0.0407)	(0.1432)
Constant	2.5078***	1.1496***	12.7776***
	(0.0338)	(0.0412)	(0.1469)
R-squared	0.043	0.067	0.291
CityFE	YES	YES	YES
YearFE	YES	YES	YES

* means *p* < 0.1, ** means *p* < 0.05, *** means *p* < 0.01, and the standard errors in parentheses.

Several mechanisms underpin these associations. First, regarding life satisfaction, the widespread application of AI technologies—ranging from smart navigation to online payment systems—has introduced conveniences that significantly enhance daily quality of life. This improvement may stimulate positive physiological responses, such as endorphin release, which helps buffer against depression (Ghosh et al., 2024). Second, in terms of social achievement, AI-driven communication tools provide older adults with richer socialization opportunities. By overcoming geographical barriers, these tools reduce social isolation and foster a sense of connection. Third, regarding cognitive level, engagement with digital devices can actively stimulate brain function, narrowing the digital divide and building cognitive resilience against mental decline. Consistent with Hopkins et al. (2023), we find that improved cognitive flexibility is strongly associated with reduced feelings of helplessness. Additionally, the integration of AI with wearable health monitoring offers a direct intervention channel. By analyzing physiological indicators such as sleep patterns, AI systems can provide personalized recommendations to improve sleep quality—a critical factor given the strong link between sleep disorders and depression in older adults. Thus, Hypothesis 2 is supported.

#### 5.1.2 Analysis of macro-level mechanism

Table 6 presents the results regarding the macro-level mediating pathways through which AI development influences depressive symptoms. The regression results indicate that the level of AI development is significantly and positively associated with the number of Internet accesses (Model 13, *p* < 0.01), the density of robot applications (Model 14, *p* < 0.01), and regional investment in science and technology (Model 15, *p* < 0.01). These findings suggest that promoting AI development is linked to significant improvements in regional digital infrastructure (num_net), automation levels (bot), and technological investment (tech). These macro-environmental factors, in turn, may contribute to alleviating depressive symptoms among older adults, confirming the mediating role of these pathways. This pattern is generally consistent with recent findings by Jiang and Luo (2024).

**Table 6:** Analysis of macro-level mechanism results.

Variables	(13)	(14)	(15)
	num_net	bot	tech
AI	0.4560***	0.6347***	0.0064***
	(0.0011)	(0.0063)	(0.0000)
age	0.0012***	−0.0026**	−0.0000
	(0.0002)	(0.0012)	(0.0000)
edu	0.0066***	−0.0068	−0.0003***
	(0.0015)	(0.0092)	(0.0000)
gender	−0.0087***	0.0182	0.0001**
	(0.0032)	(0.0190)	(0.0001)
marry	0.0012	−0.0280	0.0004***
	(0.0048)	(0.0286)	(0.0001)
family_size	−0.0038***	−0.0076	0.0001**
	(0.0011)	(0.0063)	(0.0000)
hchild	−0.0176***	0.0393***	0.0000
	(0.0014)	(0.0083)	(0.0000)
gdp	−0.0000***	−0.0000***	0.0000
	(0.0000)	(0.0000)	(0.0000)
pop	0.0004***	0.0013***	−0.0000***
	(0.0000)	(0.0001)	(0.0000)
cityrate	0.7951***	2.1673***	0.0082***
	(0.0181)	(0.1073)	(0.0004)
Constant	3.8245***	0.4330***	−0.0130***
	(0.0183)	(0.1087)	(0.0004)
R-squared	0.794	0.631	0.582
CityFE	YES	YES	YES
YearFE	YES	YES	YES

* means *p* < 0.1, ** means *p* < 0.05, *** means *p* < 0.01, and the standard errors in parentheses.

These associations can be attributed to several macroeconomic pathways. First, regional Internet accessibility serves as a critical infrastructure determining the quantity and quality of digital resources available to older adults. Higher Internet penetration fosters a more open and inclusive social environment, facilitating communication and reducing the social isolation and loneliness often linked to depression. Second, increased regional investment in science and technology creates a foundation for advanced mental health resources. The proliferation of digitized services resulting from such investment enhances accessibility for older adults, thereby lowering the probability of depression. As noted, the synergy between AI advancement and technological infrastructure creates a virtuous cycle that improves these foundational conditions. Third, a higher density of robot applications is typically indicative of greater industrial productivity and regional economic growth. As suggested by Kyu-Man et al. (2019), increased automation can lead to improved general income levels and economic security for older adults, effectively alleviating financial stress—a key risk factor for depression. Consequently, Hypothesis 3 is supported.

### 5.2 Analysis of moderating effects

Cobb–Douglas function shows that the main determinants of the level of industrial development are the amount of invested fixed assets and the amount of labor force in the case of a certain land factor (French, 2005). The higher the investment in fixed assets and the higher the number of tertiary sector employment, the higher the level of AI industry development. In order to verify whether the mitigating effect of AI industry development on the depressive symptoms of older adults is affected by the input of AI industry development in the prefecture-level city where the sample is located, this paper chooses the two variables of fixed asset investment (invest) and the number of tertiary employment (job) to characterize the moderating effect of the variables in the model. The estimation results of the moderating effects of fixed asset investment and the number of tertiary industry employment on the alleviation of depression among older adults are shown in Table 7. We can find that the coefficients of the interaction terms of the two moderating variables of fixed asset investment and the number of tertiary industry employment with the level of AI development and the coefficients of the main effects of the main effects of the relationship between the level of AI development and the depression among older adults are always consistent, and Model (16) shows that the coefficient of the interaction term between fixed asset investment and AI industry development is negative, which proves that fixed asset investment plays a positive moderating role on the main effect, fixed asset investment can strengthen the mitigating effect of AI development on the depression symptoms of older adults. Model (17) shows that the coefficient of the interaction term between the number of employment in the tertiary industry and the level of AI development is negative, which proves that the number of employees in the tertiary industry plays a positive moderating role in the main effect, the number of employees in the tertiary industry is able to strengthen the mitigating effect of AI development on the depressive symptoms of older adults. Based on the above analysis, hypothesis H4 is proved.

**Table 7:** Analysis of moderating effects results.

Variables	(16)	(17)
	cesd10	cesd10
AI	−0.0725***	−0.1363***
	(0.0165)	(0.0210)
AI*invest	−0.0000***	
	(0.0000)	
AI*job		−0.8776***
		(0.2772)
Control variables	YES	YES
	(0.2551)	(0.2846)
Constant	13.4056***	13.7750***
	(0.2647)	(0.2730)
R-squared	0.096	0.096
CityFE	YES	YES
YearFE	YES	YES

* means *p* < 0.1, ** means *p* < 0.05, *** means *p* < 0.01, and the standard errors in parentheses.

### 5.3 Heterogeneity analysis

#### 5.3.1 Urban–rural heterogeneity

**Figure 1:**
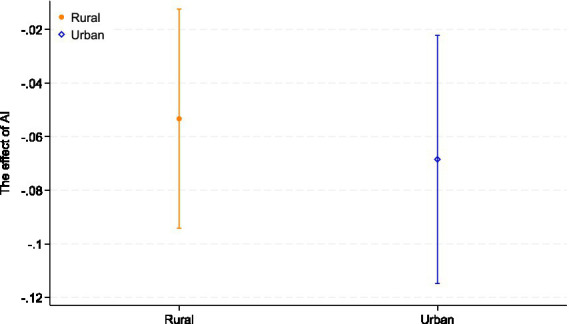
Urban–rural heterogeneity. The figure displays the coefficient estimates of AI development with 95% confidence intervals across different quantiles.

Considering that the mitigating effect of AI development on depressive symptoms in older adults may vary according to their place of residence, we divided the sample area into older adults living in urban areas and those living in rural areas for subgroup regression analysis. The results are shown in Table 8 and Figure 1. The results of Model (18) and Model (19) indicate that AI development is significantly and negatively associated with depressive symptoms among older adults in both rural areas and urban prefecture-level cities. However, a comparison of the coefficients suggests that this alleviating effect is more pronounced in urban areas compared to rural areas. This disparity can be largely attributed to the “digital divide.” Urban areas typically benefit from a higher concentration of AI technologies and mature application scenarios. In contrast, the potential of AI to alleviate depression in rural areas is constrained by weaker digital infrastructure and lower digital literacy among older adults. These structural barriers limit the effective adoption of AI-enabled services, resulting in a relatively attenuated protective effect compared to their urban counterparts.

**Table 8:** Analysis of urban–rural heterogeneity results.

Variables	(18)	(19)
	Rural	Urban
	cesd10	cesd10
AI	−0.0533**	−0.0685***
	(0.0209)	(0.0236)
age	0.0316***	−0.0053
	(0.0040)	(0.0041)
edu	−0.6460***	−0.6762***
	(0.0338)	(0.0308)
gender	−1.9580***	−1.3325***
	(0.0626)	(0.0673)
marry	−1.3843***	−1.2319***
	(0.0940)	(0.1037)
family_size	−0.0979***	−0.1050***
	(0.0188)	(0.0231)
hchild	−0.0651**	0.2436***
	(0.0265)	(0.0312)
gdp	−0.0000***	0.0000
	(0.0000)	(0.0000)
pop	−0.0001	0.0003
	(0.0002)	(0.0002)
cityrate	−2.5227***	−2.6283***
	(0.3812)	(0.3605)
Constant	12.4958***	12.2533***
	(0.3658)	(0.3905)
R-squared	0.096	0.082
CityFE	YES	YES
YearFE	YES	YES

* means *p* < 0.1, ** means *p* < 0.05, *** means *p* < 0.01, and the standard errors in parentheses.

From the perspective of ageism, these findings suggest that rural older adults may face a layered disadvantage, where structural underinvestment in digital infrastructure intersects with age-related stereotypes about limited technological competence. If AI-enabled mental health resources are mainly developed for and deployed in urban environments, older people in rural areas risk being further marginalized in both health and digital domains, reinforcing geographically patterned forms of ageism. Ensuring that AI-based mental health tools are designed and implemented in an age-inclusive way in rural communities is therefore essential to prevent the digital transformation of healthcare from widening existing inequalities in later life.

#### 5.3.2 Economic belt heterogeneity

**Figure 2:**
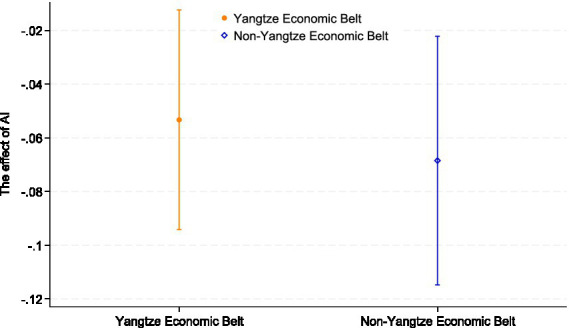
Economic belt heterogeneity. The figure plots the coefficient estimates of the interaction terms with 95% confidence intervals, illustrating the moderating effect.

AI development has a strong dependence on innovation resources. In China’s Yangtze River Economic Belt regions, the regional industrial structure is dominated by high-tech industries, and the externalities generated by these firms promote the development of the AI industry, resulting in a higher level of AI development in these regions. In contrast, in non-Yangtze River Economic Belt regions, the level of AI development is relatively sluggish due to the limited availability of innovation resources. Considering that the mitigating effect of AI development on the depressive symptoms of older adults may vary depending on the level of regional economic development, we tested the heterogeneity of innovation resources’ influence on the depressive symptoms of older adults. The results are shown in Table 9 and Figure 2. Model (20) demonstrates a significant negative association between AI development and depressive symptoms among older adults in the Yangtze River Economic Belt (YREB) region (*p* < 0.01). Conversely, Model (21) indicates no statistically significant association in non-YREB regions. This distinct regional disparity suggests that the health dividends of AI are likely contingent on the maturity of regional digital infrastructure. The YREB typically benefits from superior digital integration and resource accessibility, which facilitates the effective adoption of AI services. In contrast, the less developed infrastructure in non-YREB regions may act as a barrier, preventing AI advancements from translating into tangible alleviation of depressive symptoms for older adults. This suggests that AI development plays a more significant role in influencing the depressive symptoms of older adults living in the Yangtze River Economic Belt region. Therefore, we conclude that the effect of AI development on the depressive symptoms of older adults exhibits significant heterogeneity based on the availability of innovation resources. In order to solve the problem of unbalanced regional development, we can actively promote the flow of scientific research and medical talents, formulate industrial support measures and tax incentives, strengthen the docking of the Yangtze River Economic Belt and non-Yangtze River Economic Belt, ensure the consistency of regional development policies, narrow the development gap, and alleviate the heterogeneous impact of regional differences. However, increased reliance on AI and digital technologies could exacerbate the digital divide, especially in areas with limited internet access or advanced technology infrastructure. Older people in particular may face technological exclusion, which could lead to greater isolation from social and health services. This technological divide could limit the effectiveness of AI-driven interventions for these vulnerable populations.

**Table 9:** Analysis of economic belt heterogeneity results.

Variables	(20)	(21)
	Yangtze economic belt	Non-Yangtze economic belt
	cesd10	cesd10
AI	−0.1131***	0.1171
	(0.0165)	(0.1511)
age	0.0041	0.0464***
	(0.0030)	(0.0092)
edu	−0.8215***	−0.6412***
	(0.0235)	(0.0713)
gender	−1.5856***	−1.5030***
	(0.0484)	(0.1444)
marry	−1.3219***	−1.0162***
	(0.0738)	(0.2308)
family_size	−0.0894***	−0.1266**
	(0.0152)	(0.0552)
hchild	0.1299***	−0.1784**
	(0.0208)	(0.0741)
gdp	−0.0000***	0.0000
	(0.0000)	(0.0000)
pop	0.0003**	−0.0014
	(0.0001)	(0.0011)
cityrate	−2.1309***	−3.5848
	(0.2821)	(3.1553)
Constant	13.3267***	11.3021***
	(0.2790)	(3.8757)
R-squared	0.091	0.118
CityFE	YES	YES
YearFE	YES	YES

* means *p* < 0.1, ** means *p* < 0.05, *** means *p* < 0.01, and the standard errors in parentheses.

Viewed through the lens of ageism, the concentration of AI-related benefits in innovation-rich regions can generate a form of territorial ageism, where older adults in lagging regions are systematically excluded from emerging standards of age-friendly digital care. If policy responses focus only on boosting industrial competitiveness without explicitly addressing older adults’ access to AI-enabled services, spatial inequalities in later-life mental health may be reinforced. Recognizing equal access to AI-supported mental healthcare as a key dimension of combating ageism is therefore crucial, especially in aging societies where regional innovation gaps are pronounced.

From an ageism perspective, the concentration of AI-enabled mental health benefits in provincial capitals implies that older adults living outside these urban hubs may be doubly disadvantaged, by both age and place of residence. If high-quality AI-based services are seen as a privilege of large, technologically advanced cities, this can reinforce implicit narratives that frame digital citizenship and technological competence as attributes of younger, urban populations. Without deliberate efforts to extend age-inclusive AI infrastructures and services beyond provincial capitals, digital transformation may inadvertently entrench ageist assumptions about who is entitled to advanced mental healthcare, and widen disparities in mental health outcomes among older adults across the urban hierarchy.

#### 5.3.3 Provincial capital heterogeneity

**Figure 3:**
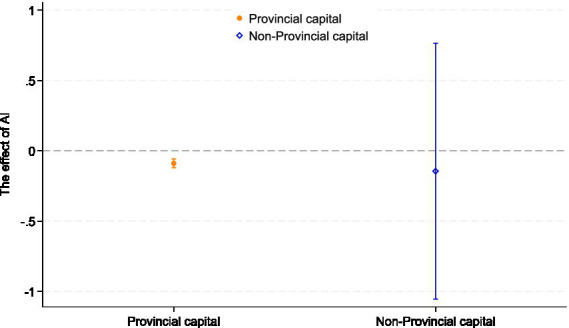
Provincial capital heterogeneity. The figure plots the coefficient estimates of the interaction terms with 95% confidence intervals, illustrating the moderating effect.

Considering that the ameliorating effect of AI development on the depressive symptoms among older adults may vary by urban location, we divided the sample into older adults living in capital cities and those living in non-capital cities, according to the Chinese government’s criteria for classifying cities. To test the possible regional heterogeneity of AI development in alleviating depressive symptoms among older adults, we conducted a group regression based on the above criteria. The results are shown in Table 10 and Figure 3. Model (22) indicates no statistically significant association between AI development and depressive symptoms in non-provincial capital cities, whereas Model (23) reveals a significant negative association in provincial capital cities (*p* < 0.01). This disparity suggests that the mental health benefits of AI are currently concentrated in major urban centers. A plausible explanation lies in the uneven distribution of digital infrastructure. Provincial capitals typically serve as regional hubs for scientific innovation and resource allocation, fostering a mature “Smart City” ecosystem. In these cities, AI applications in healthcare, transportation, and public services are more deeply integrated into daily life, providing older adults with better access to care and richer opportunities for social participation, thereby buffering against loneliness. In contrast, non-capital cities may lack the necessary digital foundation and resource density to fully translate AI potential into tangible mental health benefits for older adults, resulting in the observed lack of significant association. The logic is that provincial capital cities are usually at the forefront of scientific and technological development and application. AI technology is more commonly used in medical care, transportation, government affairs, etc. In addition, there are more cultural facilities in provincial capital cities, allowing older adults to participate more in social and cultural activities, increase the frequency of social interactions, and reduce loneliness and depression caused by social isolation. Non-provincial capital cities are at a disadvantage in these aspects. Therefore, the effect of non-provincial capital cities on depressive symptoms in older adults is relatively weaker. Therefore, we conclude that regional AI development is negatively associated with depressive symptoms among older adults exhibits significant regional heterogeneity. It is suggested that by promoting the construction of AI infrastructure in non-provincial capital areas and providing more government support, the development potential and opportunities of non-provincial capital cities can be strengthened and supplemented to improve the quality of life of older adults and alleviate depression symptoms.

**Table 10:** Analysis of regional heterogeneity results.

Variables	(22)	(23)
	Provincial capital	Non-Provincial capital
	cesd10	cesd10
AI	−0.0889***	−0.1449
	(0.0160)	(0.4639)
age	0.0077**	0.0225***
	(0.0031)	(0.0072)
edu	−0.8005***	−0.6712***
	(0.0246)	(0.0543)
gender	−1.6673***	−1.1143***
	(0.0499)	(0.1168)
marry	−1.2662***	−1.4901***
	(0.0762)	(0.1809)
family_size	−0.0968***	−0.0441
	(0.0158)	(0.0391)
hchild	0.1185***	−0.0505
	(0.0214)	(0.0571)
gdp	−0.0000***	−0.0000
	(0.0000)	(0.0000)
pop	0.0002	−0.0009
	(0.0001)	(0.0016)
cityrate	−1.8036***	−0.0916
	(0.3251)	(2.0660)
Constant	12.7929***	11.1329***
	(0.3073)	(3.1697)
R-squared	0.096	0.072
CityFE	YES	YES
YearFE	YES	YES

* means *p* < 0.1, ** means *p* < 0.05, *** means *p* < 0.01, and the standard errors in parentheses.

## 6 Conclusion

This study provides preliminary evidence that regional AI development is positively associated with better mental health among older adults in China. Using a two-way fixed-effects model, we find a significant negative association between the level of AI development in the sample areas and depressive symptoms in this population.

We further identify both macro- and micro-level pathways through which AI development may influence mental health. At the macro level, the number of Internet accesses, the density of robot applications, and investment in science and technology are all significantly related to lower depressive symptoms. At the micro level, higher life satisfaction, stronger sense of social achievement, and better cognitive functioning among older adults are also significantly associated with AI development. These pathways suggest that AI may mitigate some of the social and structural disadvantages that older adults face, including digital exclusion and unequal access to health-related resources, which are closely linked to ageism.

Finally, we observe notable heterogeneity in the mental health effects of AI development. The impact varies across residential locations and according to the availability of innovation-related resources. This pattern indicates that the benefits of AI are not evenly distributed and that emerging AI ecosystems may create new forms of inequality in later life. These findings highlight both the promise and the risks of AI for older populations and underscore the importance of designing AI-driven systems that explicitly address age-related disparities and ageist structures.

### 6.1 Enlightenments

In future work and policy practice, the transformative role of AI in mental health management for older adults warrants closer attention at both national and global levels. Our findings have several implications that are directly relevant to international debates on ageism and healthy aging.

First, interdisciplinary collaboration between AI, psychiatry, gerontology, public health, and social science is essential. Integrating AI with clinical and community-based mental health expertise could support the development of psychological assessment and rehabilitation systems that are both technologically robust and age-inclusive. Such systems should be capable of identifying universal risk factors for depression, as well as context-specific factors shaped by culture, family structures, and social norms, including internalized ageism and stigma. This approach would strengthen the theoretical basis for AI applications across diverse older populations and reduce the risk that AI tools inadvertently reproduce age-based stereotypes or diagnostic biases.

Second, international funding and cooperation mechanisms can accelerate the development and equitable diffusion of AI-driven mental health solutions. Multilateral grants and cross-border partnerships between global enterprises, local health systems, and civil society organizations could support programs that enhance digital literacy and psychological well-being among older adults. Educational initiatives that are co-designed with older adults can empower them to use AI in daily life and economic activities, while also challenging cultural narratives that depict older people as technologically incapable. Increased investment in AI research and development should therefore be accompanied by explicit safeguards against algorithmic ageism and by evaluation frameworks that consider equity in later life.

Third, addressing disparities in access to health resources and digital infrastructure for older adults in underserved regions remains a global priority. Rural areas, non–high-tech regions, and smaller cities often face compounded disadvantages: weaker infrastructure, fewer specialized professionals, and more entrenched forms of structural ageism. Expanding Internet connectivity, improving access to smart devices, and promoting age-friendly digital design in such settings could help narrow the digital divide and its mental health consequences. At the same time, policy frameworks should recognize heterogeneity within older populations and avoid one-size-fits-all solutions.

Taken together, these implications point toward a multi-tiered, globally inclusive framework for AI-enabled mental healthcare in aging societies. A stratified AI development strategy that is tailored to different social, economic, and technological contexts, and that explicitly confronts ageism, will be critical for advancing health equity in later life, supporting the Sustainable Development Goals, and improving the mental health and quality of life of older adults worldwide.

### 6.2 Limitations

Although this paper focuses on the relationship between AI development and depressive symptoms among older adults based on CHARLS data in the complex context of the intertwining of booming AI technology and aging trends in China, there are still some limitations. First, due to limitations in data availability, we were not able to obtain the names of the specific counties where the samples were located, and the granularity of the data was relatively large, which affected the representativeness of the samples to a certain extent. In addition, Self-report data may be affected by social desirability bias. When answering questions about depression, respondents may tend to provide answers that are socially acceptable or self-idealized, rather than their true psychological state. This tendency may lead to underestimation of depressive symptoms, especially in groups whose cultural background emphasizes resilience and optimism. Furthermore, potential stigma surrounding mental health issues in traditional Chinese cultural contexts may lead older adults to underreport their depressive symptoms, introducing a layer of measurement bias that implies our estimates might be lower bounds of the true association. Therefore, self-report data could be used more cautiously in future studies, and supplemented with objective measurement tools such as clinical diagnosis, behavioral observation, or physiological data when data is available. Second, this paper only selected the CHARLS database for research and analysis. The research object has certain limitations. In the future, we could focus on considering and using available data from other countries to enhance the reproducibility of the research results. Finally, although the mediation analysis illuminates pathways linking AI use to depressive symptoms, the observational CHARLS data limits causal inference. While we employ instrumental variables to mitigate endogeneity, unobserved confounders may still bias results, rendering findings suggestive rather than definitive. More reliable causal inference results may be obtained in the future using Randomized Controlled Trials or natural experiments. Finally, although we employed instrumental variable strategies to mitigate endogeneity, our data remains observational. Therefore, causal interpretations should be made with caution. Future studies using longitudinal designs or natural experiments are needed to further validate the causal impact of AI on mental health.

## Data Availability

The original contributions presented in the study are included in the article/supplementary material, further inquiries can be directed to the corresponding authors.

## Footnotes

1
http://charls.pku.edu.cn/
